# Chrysomycin A Attenuates Neuroinflammation by Down-Regulating NLRP3/Cleaved Caspase-1 Signaling Pathway in LPS-Stimulated Mice and BV2 Cells

**DOI:** 10.3390/ijms22136799

**Published:** 2021-06-24

**Authors:** Man Liu, Shan-Shan Zhang, Dong-Ni Liu, Ying-Lin Yang, Yue-Hua Wang, Guan-Hua Du

**Affiliations:** 1 State Key Laboratory of Bioactive Substance and Function of Natural Medicines, Institute of Materia Medica, Chinese Academy of Medical Sciences & Peking Union Medical College, Beijing 100050, China; liuman@imm.ac.cn (M.L.); zhangshanshan@imm.ac.cn (S.-S.Z.); liudongni@imm.ac.cn (D.-N.L.); yinglin@imm.ac.cn (Y.-L.Y.); 2 Beijing Key Laboratory of Drug Target Identification and New Drug Screening, Institute of Materia Medica, Chinese Academy of Medical Sciences & Peking Union Medical College, Beijing 100050, China

**Keywords:** Chrysomycin A, neuroinflammation, BV2 microglia cells, lipopolysaccharide (LPS)

## Abstract

Chrysomycin A (Chr-A), an antibiotic chrysomycin, was discovered in 1955 and is used to treat cancer and tuberculosis. In the present study, the anti-neuroinflammatory effects and possible mechanism of Chr-A in BALB/c mice and in BV2 microglia cells stimulated by lipopolysaccharide (LPS) were investigated. Firstly, the cortex tissues of mice were analyzed by RNA-seq transcriptome to identify differentially expressed genes (DEGs) regulated by Chr-A in LPS-stimulated mice. Inflammatory cytokines and inflammatory proteins were detected by enzyme-linked immunosorbent assay and Western blot. In RNAseq detection, 639 differential up-regulated genes between the control group and LPS model group and 113 differential down-regulated genes between the LPS model group and Chr-A treatment group were found, and 70 overlapping genes were identified as key genes for Chr-A against neuroinflammation. Subsequent GO biological process enrichment analysis showed that the anti-neuroinflammatory effect of Chr-A might be related to the response to cytokine, cellular response to cytokine stimulus, and regulation of immune system process. The significant signaling pathways of KEGG enrichment analysis were mainly involved in TNF signaling pathway, cytokine–cytokine receptor interaction, NF-κB signaling pathway, IL-17 signaling pathway and NOD-like receptor signaling pathway. Our results of in vivo or in vitro experiments showed that the levels of pro-inflammatory factors including NO, IL-6, IL-1β, IL-17, TNF-α, MCP-1, CXCL12, GM-CSF and COX2 in the LPS-stimulated group were higher than those in the control group, while Chr-A reversed those conditions. Furthermore, the Western blot analysis showed that its anti-neuroinflammation appeared to be related to the down-regulation of NLRP3/cleaved caspase-1 signaling pathway. The current findings provide new insights into the activity and molecular mechanisms of Chr-A for the treatment of neuroinflammation.

## 1. Introduction

Neuroinflammation has been regarded as having a potential role in the progression or etiology of various central nervous system (CNS) diseases, including cerebral ischemia-reperfusion injury, Alzheimer’s disease (AD) and Parkinson’s disease (PD) [1,2,3]. Microglia cells, as the central effectors of neuroinflammation, provide structural and metabolic support in the brain and participate in clearing cellular debris through phagocytosis [4]. Microglia express numerous pattern recognition receptors and can be activated by LPS, a vital molecular component of the cell outer membrane of Gram-negative bacteria, via up-regulating Toll-like receptors (TLRs) and NOD-like receptors (NLRs) signaling pathways, followed by the production and release of pro-inflammatory cytokines [5,6]. Recently, inflammasomes have emerged as a critical factor in neuroinflammation and a series of related diseases [7]. Among multiple inflammasomes, NLRP3 is widely known to trigger the activation of caspase-1 and then promote the maturation of interleukin-1β (IL-1β) and interleukin-18 (IL-18) [8,9]. IL-1β and IL-18 drive inflammatory responses by initiating downstream signaling pathways, leading to neuronal damage or death [10]. IL-1β and IL-18 have been found with high levels in the cerebrospinal fluid (CSF) of patients with neurodegenerative diseases [11,12]. Thus, targeting the NLRP3/cleaved caspase-1/IL-1 axis has been a therapeutic strategy for neurological diseases. Most exogenous compounds that anti-IL-1 and inhibit the activation of the NLRP3 inflammasome are in the early stages of development [13].

**Figure 1:**
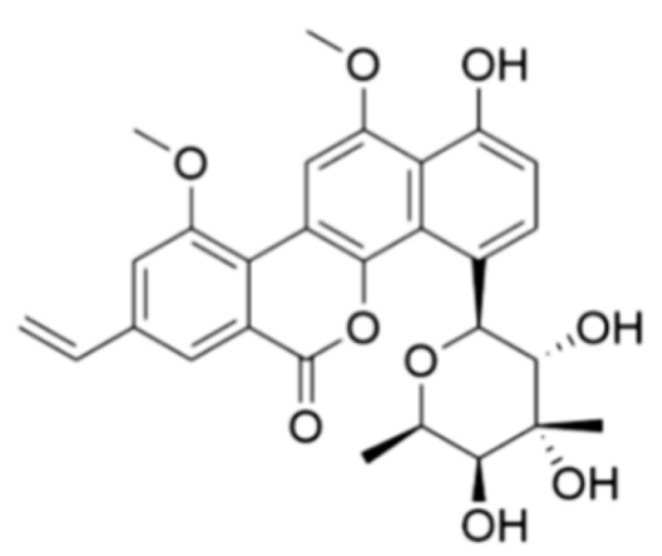
Structure of Chrysomycin A.

Chrysomycin A (Chr-A) (Figure 1) is a yellow, crystalline antibiotic chrysomycin that was discovered in 1955 by Strelitz et al. [14]. Chr-A was reported to be active against a panel of cancer cell lines and had anti-tumor activity [15,16]. In a recent study, Chr-A was demonstrated to have anti-tuberculosis activity against multi-drug-resistant (MDR) and extreme-drug-resistant (XTR) TB strains [17]. However, the ability of Chr-A in anti-neuroinflammation is rarely reported. Given these findings, in this study, we explored the anti-neuroinflammation effects and the possible mechanism of Chr-A on LPS-induced injury in vivo and in vitro.

## 2. Results

### 2.1. Identifying Differential Expression Genes after Chr-A Treatment in LPS-Stimulated Mice by RNA-Seq Transcriptome Analysis

**Figure 2:**
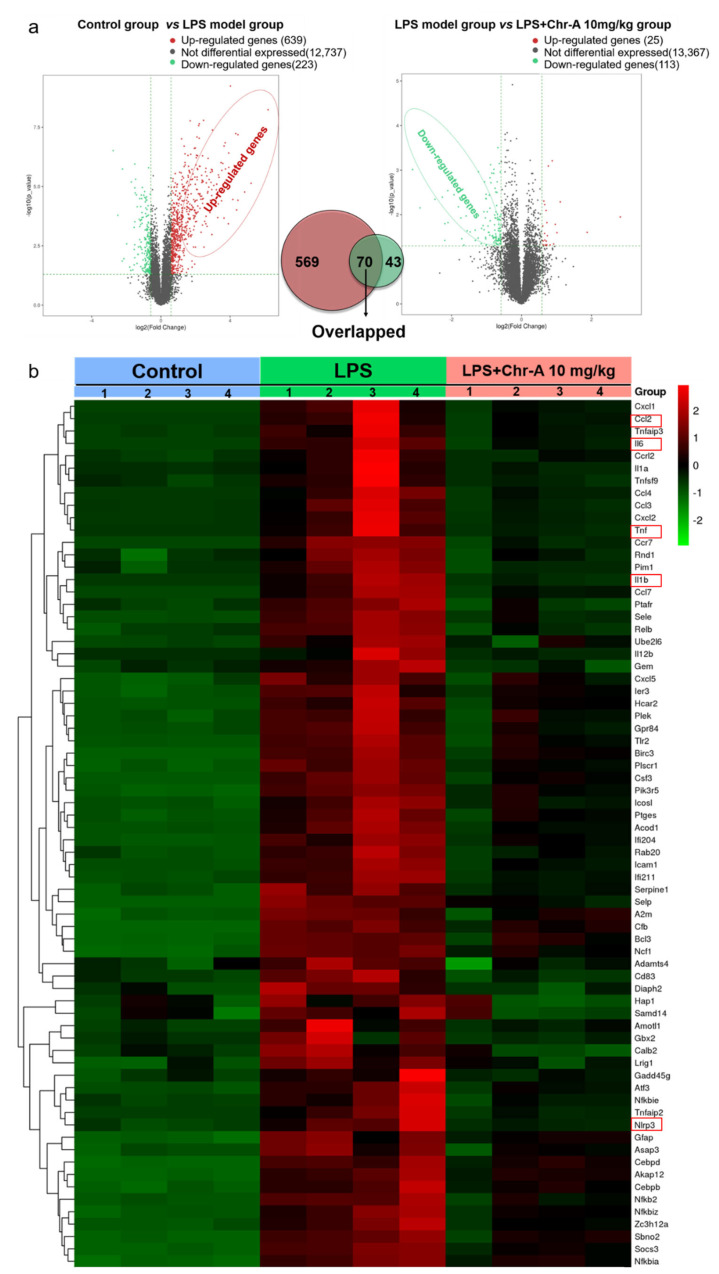
RNA sequencing analysis of differential genes. Volcano map analysis of all DEGs in control group, LPS model group and LPS + Chr-A 10 mg/kg group (*n* = 4) (**a**). Seventy overlapping genes of three groups are shown in the heat map. In this diagram, red represents higher expression and green represents lower expression (**b**).

In order to reveal the crucial targets for protective effect of Chr-A for treatment of LPS-stimulated neuroinflammation, RNA sequencing analysis was conducted among the control group, LPS model group and an LPS + Chr-A 10 mg/kg group. Firstly, differential expression genes (DEGs) between the control group and the LPS model group were screened. The results showed that 639 differential up-regulated genes and 223 differential down-regulated genes were obtained. Then, DEGs between the LPS model group and the Chr-A treated group were screened, and 25 differential up-regulated genes and 113 differential down-regulated genes were found. Notably, 70 genes were identified both in the up-regulated genes of the control group vs. the LPS model group and in the down-regulated genes of the LPS model group vs. the Chr-A treatment group (Figure 2a). As shown in the heat map, the 70 overlapping genes were clearly recognized by different colors among these three groups (Figure 2b). Furthermore, these 70 overlapping genes were regarded as the core genes for Chr-A against neuroinflammation followed by GO and KEGG pathway enrichment analysis.

### 2.2. Enrichment Analysis of Core Genes for Chr-A against Neuroinflammatory

**Figure 3:**
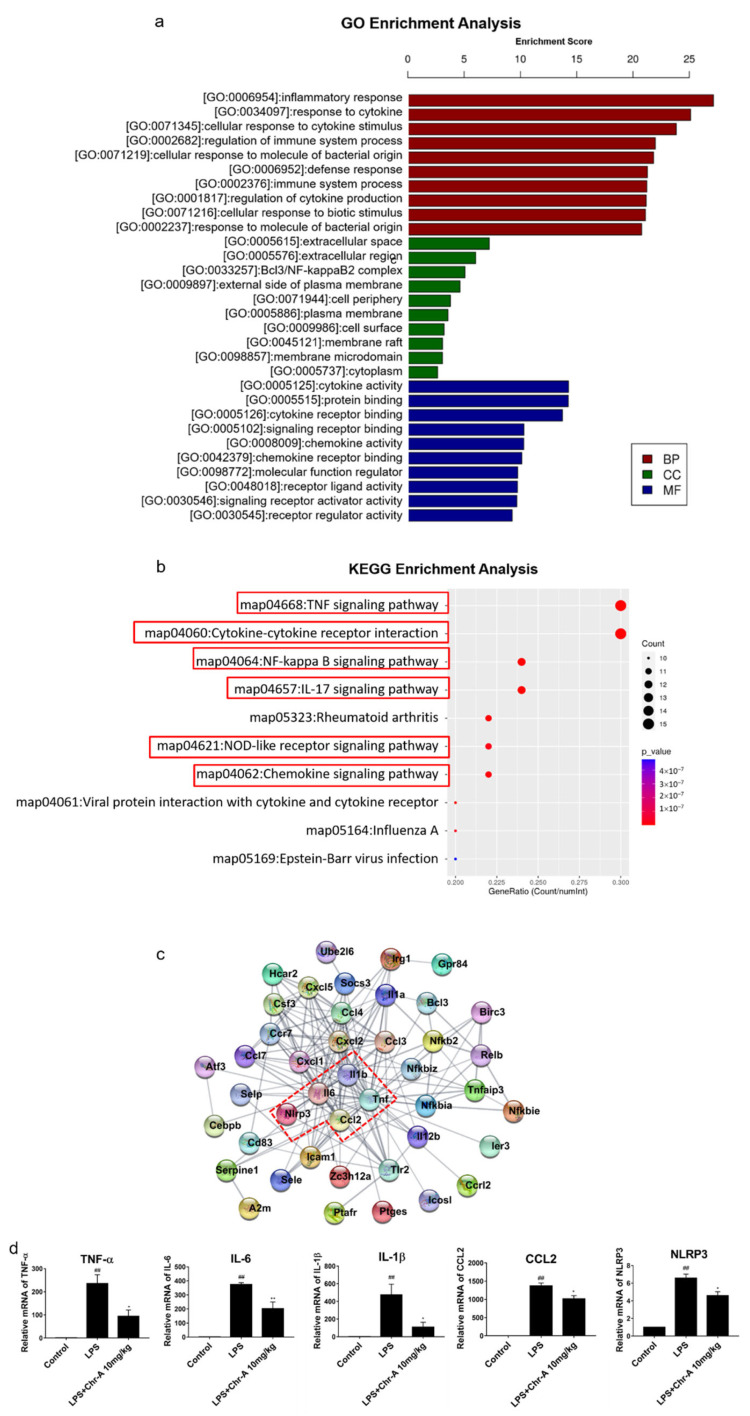
GO and KEGG enrichment analysis of core genes for Chr-A against neuroinflammation. Top 10 significant GO terms are divided into BP, CC, and MF (**a**). Top 10 significant KEGG pathways (**b**). PPI network of core genes for Chr-A against neuroinflammatory (**c**). Relative mRNA level of IL-6, TNF-α, IL-1β, CCL2 and NLRP3 (*n* = 3) (**d**). Data are expressed as the mean ± SEM. ## *p* < 0.01 vs. control group; * *p* < 0.05, ** *p* < 0.01 vs. LPS model group.

To reveal the underlying pharmacological mechanisms of Chr-A against neuroinflammation, the 70 overlapping genes were analyzed for GO and KEGG enrichment analysis. The top ten significant GO terms, including biological process (BP), cellular component (CC) and molecular function (MF) (Figure 3a), and top ten significant KEGG pathways (Figure 3b) are listed. The GO enrichment results indicated that the biological process of Chr-A against neuroinflammatory were related to inflammatory response, response to cytokine, cellular response to cytokine stimulus, regulation of immune system process and cellular response to molecular of bacterial origin; cellular components of Chr-A against neuroinflammation were related to extracellular space, extracellular region, Bcl3/NF-κB2 complex, external side of the plasma membrane and cell periphery; and molecular function of Chr-A for treatment neuroinflammatory include cytokine activity, protein binding, cytokine receptor binding, signaling receptor binding and chemokine activity. Furthermore, the significant signaling pathways of KEGG enrichment analysis were mainly involved in the TNF signaling pathway, cytokine–cytokine receptor interaction, NF-κB signaling pathway, IL-17 signaling pathway, NOD-like receptor signaling pathway and chemokine signaling pathway.

In addition, the core genes of Chr-A against neuroinflammatory response are not isolated but interconnected, so the PPI network of 70 core genes was constructed and analyzed in this study to identify the hub proteins. As illustrated in Figure 3c, after excluding disconnected nodes in the network, the TNF-α, IL-6, IL-1β and CCL2 ranked the top among all the included proteins with the degree value at 29, 23, 23 and 16, respectively. Furthermore, according to the results of KEGG enrichment analysis, TNF-α, IL-6, IL-1β and CCL2 were also closely related to TNF signaling pathway, cytokine–cytokine receptor interaction, NF-κB signaling pathway, IL-17 signaling pathway, NOD-like receptor signaling pathway and chemokine signaling pathway. Moreover, NLRP3 is one of the most important proteins in the NLRP3/cleaved caspase-1 pathway that we were strongly interested in the present study. Therefore, we further performed RT-PCR analysis of TNF-α, IL-6, IL-1β, CCL2 and NLRP3 of the cortex tissue, and the results of RT-PCR shown in Figure 3d were consistent with RNA-Seq analysis.

### 2.3. Chr-A Inhibits Neuroinflammation in the Cortex Tissues of LPS-Induced Mice

**Figure 4:**
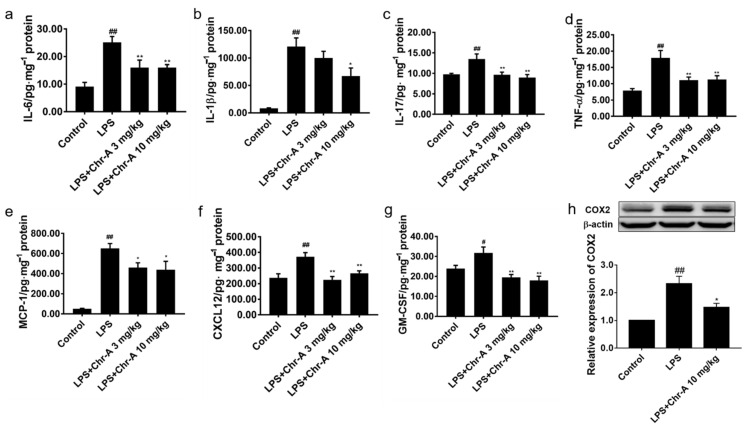
Effect of Chr-A on pro-inflammatory factors, chemokines and inflammation related protein the cortex of mice stimulated by LPS. The levels of IL-6 (**a**), IL-1β (**b**), IL-17 (**c**), TNF-α (**d**), MCP-1 (**e**), CXCL12 (**f**) and GM-CSF (**g**) were assessed by ELISA (*n* = 7–8). The expression level of COX2 (**h**) were assessed by Western blot (*n* = 5). Data are expressed as the mean ± SEM. # *p* < 0.05, ## *p* < 0.01 vs. control group; * *p* < 0.05, ** *p* < 0.01 vs. LPS model group.

After LPS 5 mg/kg injection, the body temperature of BALB/c mice was decreasing and the mice appeared tremor and squinting, indicating that a systematic inflammatory model was conducted. As shown in Figure 4a–g, the levels of pro-inflammatory cytokine and chemokines in the cortex of LPS group were significantly higher than those in the control group. However, Chr-A at the doses of 3 and 10 mg/kg reduced the pro-inflammatory cytokine and chemokines levels, including IL-6, IL-1β, IL-17, TNF-α, GM-CSF, CXCL12 and MCP-1. Furthermore, LPS stimulation significantly increased the levels of COX2 on the protein expression in the cortex of mice. However, 10 mg/kg Chr-A administration inhibited the expression of COX2 (Figure 4h) in mice.

### 2.4. Chr-A Reduced Inflammatory Factors Production in the Supernatants of LPS-Stimulated BV2 Microglia Cells

**Figure 5:**
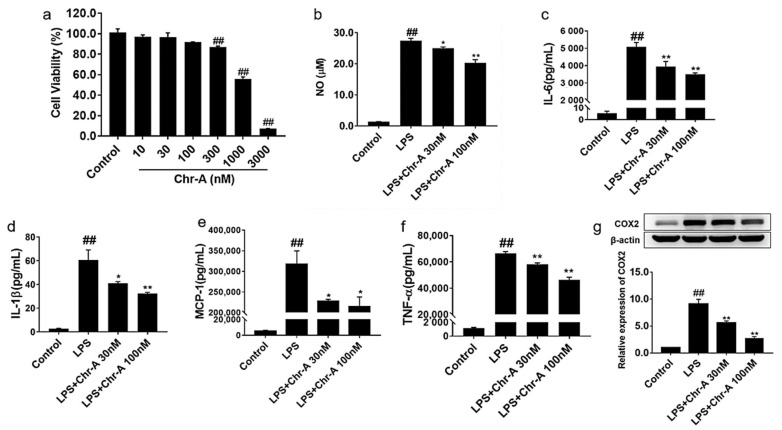
Effect of Chr-A on pro-inflammatory factors in the supernatants of BV2 cells. Cell viability of Chr-A on BV2 microglia cells (**a**). The effect of Chr-A on NO production (**b**). The levels of IL-6 (**c**), IL-1β (**d**) MCP-1 (**e**) and TNF-α (**f**) were assessed by ELISA. The expression level of COX2 was assessed by Western blot (**g**). LPS stimulating time was 24 h for above-mentioned analysis. Data are expressed as the mean ± SEM. Experiments were performed in triplicate. ## *p* < 0.01 vs. control group; * *p* < 0.05, ** *p* < 0.01 vs. LPS model group.

To examine the toxicity of Chr-A on BV2 microglia cells, a series of concentrations of Chr-A (10, 30, 100, 300, 1000 and 3000 nM) and BV2 cells were incubated together for 24 h, and CCK8 analysis was conducted. As shown in Figure 5a, the cell viability at concentrations of 10, 30 and 100 nM had no significant change. Therefore, 30 and 100 nM Chr-A were used for all subsequent experiments. LPS is well known to activate microglia and promote the release of various inflammatory factors. We further investigated the levels of inflammatory cytokines and chemokine inthe supernatants of LPS-induced BV2 microglia cells. LPS could promote the release of NO in the BV2 microglia cells and thus activate microglia. In Figure 5b, 200 ng/mL LPS induction for 24 h significantly increased the levels of NO in the supernatants of BV2 cells compared with the control group. After treatment with Chr-A for 24 h, 30 and 100 nM Chr-A significantly decreased the levels of NO. The levels of inflammatory cytokines IL-6, IL-1β and TNF-α and chemokine MCP-1 were detected by ELISA. The results showed that LPS induced the release of IL-6, IL-1β MCP-1 and TNF-α in microglia. However, Chr-A dose-dependently decreased the production of pro-inflammatory factors in the supernatants of LPS-induced BV2 microglia cells as shown in Figure 5c–f. As shown in Figure 5g, Western blot assay showed that LPS stimulation significantly increased the protein expression levels of COX2 in BV2 cells. However, treatment with 30 and 100 nM Chr-A inhibited the protein expression of COX2 in LPS-induced BV2 cells.

### 2.5. Chr-A Down-Regulated NLRP3/Cleaved Caspase-1 Signaling Pathway in LPS-Induced Injury Both In Vivo and In Vitro

**Figure 6:**
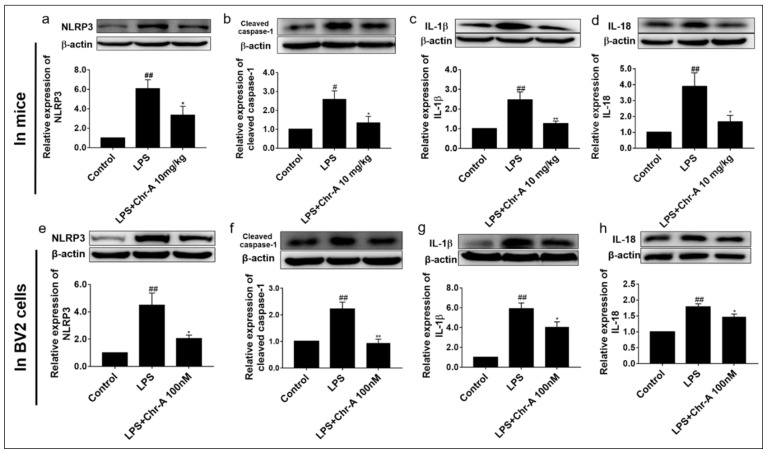
Effect of Chr-A on NLRP3/Cleaved caspase-1 signaling pathway. Protein expression levels of NLRP3 (**a**), cleaved caspase-1 (**b**), IL-1β (**c**) and IL-18 (**d**) in BALB/c mice (*n* = 5). Protein expression levels of NLRP3 (**e**), cleaved caspase-1 (**f**), IL-1β (**g**) and IL-18 (**h**) in BV2 microglia cell. Experiments were performed in triplicate. LPS stimulating time was 24 h for NLRP3, cleaved caspase-1 and IL-1β Western blot analysis and 12 h for IL-18 Western blot analysis. Data are expressed as the mean ± SEM, # *p* < 0.05, ## *p* < 0.01 vs. control group; * *p* < 0.05, ** *p* < 0.01 vs. LPS model group.

Nlrp3 gene was identified remarkably by RNA-seq, and the KEGG enrichment results indicated that the NOD-like receptor signaling pathway was significantly enriched. Therefore, we evaluated the protein expression of NLRP3/cleaved caspase-1 signaling pathway both in vivo and in vitro. We found that LPS stimulation significantly increased the levels of NLRP3, cleaved caspase-1 and downstream IL-1β and IL-18 on the protein expression in the cortex of mice. However, 10 mg/kg Chr-A administration inhibited the expression of NLRP3, cleaved caspase-1, IL-1β and IL-18 (Figure 6a–d). Western blot assay results also showed that LPS significantly increased the protein expression of NLRP3, cleaved caspase-1, IL-1β and IL-18 in BV2 cells. However, treatment of 100 nM Chr-A inhibited the protein expression of NLRP3, cleaved caspase-1, IL-1β and IL-18 in BV2 microglia (Figure 6e–h), which was consistent with the data of mice.

## 3. Discussion

Since Strelitz et al. found Chrysomycin A (Chr-A), a yellow crystalline antibiotic chrysomycin, in 1955, it has been demonstrated to have anti-tumor and anti-tuberculosis activity [14,15,16]. To our knowledge, this is the first report investigating whether Chr-A could inhibit LPS-induced neuroinflammation and its possible mechanism. In this study, we investigated the effects and mechanism of Chr-A against neuroinflammation both in vivo and in vitro. The results indicated that Chr-A attenuated LPS-induced neuroinflammation, and the possible mechanisms might relate to down-regulating the NLRP3/cleaved caspase-1 inflammatory pathway.

Neuroinflammation, a specialized immune response that takes place in the CNS, has been reported to link to stroke, traumatic brain injury and neurodegenerative diseases, such as AD and PD [18,19,20]. LPS is one of immune-stimulatory components of Gram-negative bacteria and Lipid A is the main pathogen-associated molecular pattern (PAMPs) of LPS [21]. LPS injection is a common method to perform an inflammatory response model and is well reproduced [22]. After being recognized, especially by TLR4, LPS triggers inflammatory and immune responses. Our previous investigation showed that HMGB1/TLR4/MyD88 signaling pathway was activated after LPS stimulation in the striatum and cortex of LPS-induced mice [23,24,25,26]. According to RNA-sequencing analysis, we found 70 overlapping genes that were up-regulated after LPS stimulating and down-regulated when treated with 10 mg/kg Chr-A in mice. KEGG pathway analysis of these genes indicated that TNF signaling pathway, cytokine–cytokine receptor interaction, NF-κB signaling pathway, IL-17 signaling pathway, NOD-like receptor signaling pathway and chemokine signaling pathway might be the major pathways of Chr-A treatment for neuroinflammation injury.

It is well known that microglia, as the first immunological barrier, plays an important role in neuroinflammation to defense pathogens and environmental insults [27]. Excessive inflammatory responses lead to the activation of microglia, following the release of pro-inflammatory factors, including IL-1β, IL-6 and TNF-α [28]. These pro-inflammatory factors aggravate neuroinflammatory reactions and cause neuronal cell degeneration in turn [29]. Studies have indicated that pro-inflammatory cytokines/chemokines released from activated microglia were important mediators in sepsis-associated brain inflammation [30]. In the present study, we found that LPS induced the release of pro-inflammatory cytokines, such as IL-1β, IL-6, IL-17 and TNF-α; chemokines, such as MCP-1 and CXCL12; and colony-stimulating factor, such as GM-CSF in the cortex of LPS-induced mice. However, Chr-A treatment decreased the levels of these inflammatory factors in cortex tissue. Similarly, LPS exposure resulted in the increase in pro-inflammatory factors IL-1β, IL-6, MCP-1, TNF-α and NO in BV2 microglial cells, and Chr-A inhibited the levels of these inflammatory factors. Over the past two decades, it has been reported that COX2 was a central signaling molecule for many inflammatory processes. Microglia activation results in COX2 induction and leads to the accumulation of PGE2 and other inflammatory mediators in themselves as well as neurons, which subsequently affect neuronal and brain function [31]. It is reported that the administration of COX2 inhibitors suppresses the inflammatory cascade and benefits behavioral recovery in some neurodegenerative diseases [32]. In this study, the data demonstrated that in comparison with the control group, COX2 level was higher in the LPS stimulated group, and Chr-A administration reduced the LPS-induced elevation of COX2 both in mice and BV2 cells. These results indicated that Chr-A could protect the brain from neuroinflammation both in vivo and in vitro.

Cytokines and chemokine participate and play a vital role in inflammatory procedures. Chemokines, a kind of small chemoattractant peptide, provide directional cues for cell trafficking and therefore recruit leukocytes to the site of inflammation of foreign insult [33,34]. Cytokines are crucial intercellular regulators and mobilizers for cells engaging in innate as well as adaptive inflammatory host defenses. They are released by various cells in response to an activating stimulus and trigger the following responses via binding to specific receptors on the cell surface of target cells [35]. The nuclear factor-κB (NF-κB) signaling pathway is a classical signaling transduction pathway of both innate and adaptive immunity. As NF-κB signaling pathway-related pathways, TNF signaling pathway and IL-17 signaling pathway initiate the transcription of downstream inflammatory factors via activating the NF-κB signaling pathway and also play a crucial role in the inflammatory response [36,37]. After activating by inducers, the inhibitors of NF-κB (IκB) become phosphorylated, ubiquitylated and then degraded by proteasome. Then, NF-κB translocates from cytoplasm to the nucleus after the degradation of IκB and binds their cognate DNA binding sites, starting the transcription of many genes, including cytokines, chemokines and stress-response proteins [38]. Inflammasomes are cytosolic multiprotein complexes mainly expressed in immune cells and are responsible for the detection and elimination of pathogen-associated molecular pattern (PAMPs) and damage-associated molecular pattern (DAMPs). Among different inflammasomes discovered so far, NLRP3 inflammasome is the most fully characterized inflammasome assembled by the NLRP3 scaffold, the ASC (PYCARD) adaptor, and caspase-1 [9]. Some studies have shown that NLRP3 inflammasome has been involved in many neurological diseases including chronic neurodegenerative disease [39]. Activation of NLRP3 inflammasome involves two steps, priming and activation. The priming step is conferred by the stimulation of TLRs by exposure to PAMPs or DAMPs and subsequently results in NF-κB signaling pathway activation. Then, NF-κB induces transcription and the expression of NLRP3, pro-IL-1β, and pro-IL-18, which remain inactive in cytoplasm. In the activation step, a series of subsequent stimulus, such as K^+^ efflux, the generation of ROS and the release of rupturing lysosomal contents, activate the NLRP3 inflammasome by promoting the oligomerization of NLRP3, ASC and pro-caspase-1. Then, activated NLRP3 inflammasome catalyzes the conversion of pro-caspase-1 to the active cleaved caspase-1, which contributes to activation, maturation and up-regulation of IL-1β and IL-18 pro-inflammatory cytokines [8]. IL-1β and IL-18, in turn, stimulate multiple signaling pathways and drive inflammatory responses, which results in neuronal injury or death [40]. Given the complex of NLRP3 inflammasome signaling pathways mentioned above, it is not surprising that NLRP3 inflammasome has been proven to play an important role in multiple inflammatory diseases, not only immune-inflammatory but also neuroinflammation and metabolic inflammation [41]. Studies showed that in NLRP3^−/−^ and Casp-1^−/−^ mice, memory deficits and neurobehavioral disturbances were alleviated and the deficiency of NLRP3 reduced LPS-elicited increasing of IL-1βin mice [42,43]. All these findings highlighted the promising application of NLRP3 inflammasome signaling pathway inhibition in various neuroinflammatory-related disorders. In the present study, NLRP3, cleaved caspase-1 and the downstream proteins, IL-1β and IL-18, were all up-regulated in the LPS model group compared with the control group in the cortex of mice, suggesting that NLRP3/cleaved caspase-1 signaling pathway was activated after LPS stimulation. However, Chr-A administration significantly decreased the expression of NLRP3, cleaved caspase-1, IL-1β and IL-18. Similarly to the data obtained from the LPS-induced mice, Chr-A treatment also inhibited the expression of NLRP3, cleaved caspase-1, IL-1β and IL-18 in LPS-induced microglia. However, the NLRP3 inflammasome-related signaling pathways are quite complex, and we still need further experiments to reveal the in-depth mechanism on how Chr-A down-regulating NLRP3/cleaved caspase-1 signaling pathway. Whether Chr-A regulating NLRP3/cleaved caspase-1 signaling pathway by directly interacting with NLRP3 and inhibiting the assembling of NLRP3 inflammasome complex was not discussed in this study. Thus, in the future, different specific inhibitors, such as MCC950 (selective NLRP3 inhibitor), would be utilized to explore the in-depth mechanism of Chr-A.

## 4. Materials and Methods

### 4.1. Regents

Chrysomycin A was provided by Prof. Hua-Wei Zhang (Zhejiang University of Technology). Dulbecco’s modified Eagle’s medium (DMED) and fetal bovine serum (FBS) for cell culture were purchased from Gibco BRL (Grand Island, NY, USA). LPS (Escherichia coli 0127: B8) were obtained from Sigma-Aldrich (St. Louis, MO, USA). ELISA kits IL-1β (EM001), IL-6 (EM004), TNF-α (EM008), MCP-1 (EM018) and GM-CSF (EM020) were purchased from ExCell Biology (Shanghai, China). IL-17 (PI545), and CXCL12 (PC201) were purchased from Beyotime Biotechnology (Shanghai, China). Antibodies against COX2 (ab15191) and IL-18 (ab207323) were purchased from Abcam (Cambridge, UK). Antibodies for β-actin (3700), NLRP3 (15101), cleaved caspase-1 (89332) and IL-1β (12242) were purchased from Cell Signaling Technology (Beverley, CA, USA).

### 4.2. Experimental Procedure of LPS-Stimulated Mice

Adult male BALB/c mice (18–22 g) were purchased from SPF (Beijing) Biotechnology Co., Ltd. (Beijing, China; Animal certification number was SCXK (Jing) 2019-0010). All animals were housed in a pathogen-free facility with 12 h of light and dark per day at an ambient temperature of 23 ± 2 °C and humidity of 55 ± 5%. BALB/c mice were acclimatized for 3 days prior to experimentation and then randomly divided into the following four groups: control group (Control), pre-treatment with an equal volume of vehicle (normal saline containing 0.1% Tween 80) for 7 days and normal saline by i.p. on the 7th day; LPS model group (LPS), pre-treatment with an equal volume of vehicle for 7 days and LPS (5 mg/kg) by i.p. on the 7th day; Chr-A treatment groups, pre-treatment with Chr-A at doses of 3 mg/kg (LPS + Chr-A 3 mg/kg) and 10 mg/kg (LPS + Chr-A 10 mg/kg) individually by *i.p*. for 7 days and LPS (5 mg/kg) by i.p. on the 7th day. Six hours after LPS stimulation, the cortex of mice was removed and frozen at −80 °C for further research [23,24,26]. All animal care and experimental procedures regarding the animals were approved by the ethic committees of the Institute of Materia Medica, Chinese Academy of Medical Sciences and Peking Union Medical College.

### 4.3. RNA-Seq and Enrichment Analysis

The cortex tissues of control, LPS model and LPS + Chr-A 10 mg/kg groups were processed for RNA-seq transcriptome analysis using Hiseq4000 (Illumina, Hayward, CA, USA). The raw sequences were processed for quality control, trimming (Trimmomatic v. 0.36) and alignment (STAR/2.5.1b). Downstream analyses were performed by using R (v.3.1.1), with edgeR and limma package with the voom method. Differentially expressed genes (DEGs) were further conducted for GO and KEGG pathway significant enrichment analysis [1].

### 4.4. Protein–Protein Interaction (PPI) Network Construction of Core Targets

The core targets were introduced into String (https://string-db.org/, accessed on 1 March 2021), a database that provides protein–protein interactions and functional associations, to obtain the interactions between these proteins. The species was set as “Mus Musculus”, the minimum required interaction score was set as “High confidence (0.700)” and the disconnected nodes in the network were hidden. Then, the PPI network was diagrammed via Cytoscape 3.8.0 software.

### 4.5. BV2 Microglia Cells Culture and Treatment

BV2 microglia cells were purchased from Cell Resource Center, Institute of Basic Medical Sciences, Chinese Academy of Medical Sciences and Peking Union Medical College (Beijing, China) and maintained in DMED and 10% fetal bovine serum at 37 °C in a 5% CO_2_ incubator. BV2 microglia cells were cultured in a 96-well plate (2.5 × 10^4^ cells/well) or 6-well plates (1 × 10^6^ cells/well). At about 80% confluence, BV2 cells were pre-incubated with Chr-A 30 and 100 nM for 1 h and then exposed to 200 ng/mL LPS for another 24 h or 12 h [25].

### 4.6. NO Determination in the Supernatant of BV2 Cells

The level of NO was determined by assaying the concentration of nitrite in the cell supernatant by the Griess reaction using NO assay kit (Applygen Technologies Inc., Beijing, China) according to the manufacturer’s instructions [25].

### 4.7. Inflammatory Factors Determined by ELISA Assay

The levels of IL-6, IL-1β, MCP-1 and TNF-α in the supernatant of BV2 cells were determined using ELISA kits. The supernatant of cortex tissues was prepared as described previously [24], and then the levels of IL-6, IL-1β, TNF-α, MCP-1, IL-17, GM-CSF and CXCL12 were detected by ELISA kits according to the introductions of the manufacturer.

### 4.8. Real-Time PCR Analysis

Total RNA was extracted from cortex tissues of BALB/c mice using Trizol reagent (Ambion, Carlsbad, CA, USA) according to the manufacturer’s instructions [25]. The RNA samples (1000 ng/μL) were used for reverse transcription with MonScriptTMRTⅢ All-in-One Mix (Monad Biotech Co., Ltd., Wuhan, China). The primers included β-actin (forward, AGGCCAACCGTGAAAAGATG; reverse, TGGCGTGAGGGAGAGCATAG), NLRP3 (forward, TGGATGGGTTTGCTGGGAT; reverse, CTGCGTGTAGCGACTGTTGAG), IL-6 (forward, CCACTTCACAAGTCGGAGGCTTA; reverse, GCAAGTGCATCATCGTTGTTCATAC), IL-1β (forward, CCAGGATGAGGACATGAGCA; reverse, CGGAGCCTGTAGTGCAGTTG) and TNF-α (forward, CCACGCTCTTCTGTCTACTG; reverse, ACTTGGTGGTTTGCTACGAC) and CCL2 (forward, AGCACCAGCACCAGCCAACT; reverse, CAGGTGACTGGGGCATTGAT). Then, according to the primers, 2 μL cDNA templates were subjected to real-time PCR using SYBR^®^ qPCR Master Mix (Vazyme Biotech Co., Ltd., Nanjing, China). PCR was performed using the following protocol: 5 min at 95 °C, followed by 40 cycles of 10 s at 95 °C, 30 s at 60 °C, and 15 s at 95 °C, 60 s for 60 °C and 15 s for 95 °C. According to the relative quantification of 2^−^^∆∆Ct^ method, the transcription expression of target genes could be determined when using β-actin as internal reference.

### 4.9. Western Blot Assay

After treatment with Chr-A, the cortex tissues of in LPS-stimulated mice or the BV2 cells after LPS-stimulated were collected and lysed by Radio Immunoprecipitation Assay (RIPA) buffer in the presence of cocktail protease inhibitors (Thremo, Waltham, MA, USA) on ice for 30 min and then centrifuged at 4 °C at 12,000× *g* for 15 min. The supernatant was taken, and the protein concentration was determined using BCA assay kit (Thremo, USA). Then, loading buffer was added and boiled for 10 min at 100 °C. Total protein was separated by 10–15% SDS-PAGE, and the protein bands were transferred to polyvinylidene fluoride (PVDF) membrane. The membranes were blocked by incubation with 5% bovine serum albumin (BSA) in 1× TBST buffer (10 mM Tris-HCl, 150 mM NaCl, and 0.5% Tween-20) for 2 h at room temperature and then incubated with different primary antibodies: anti-COX2 antibody (Rt, 1:1000), anti-NLRP3 antibody (Rt, 1:1000), anti-cleaved caspase-1 antibody (Rt, 1:1000), anti-IL-1β antibody (Ms, 1:1000), anti-IL-18 antibody (Rb, 1:1000) and anti-β-actin (Ms, 1:3000) overnight at 4 °C. After washing with 1× TBST, the membranes were incubated with HRP-conjugated secondary antibody for 2 h at room temperature followed by washing and finally detected by enhanced ECL system. The signal densities on the blots were measured by Gel-pro software (Molecular Imager ChemiDoc XRS+System, Bio-Rad, Irvine, CA, USA) and normalized by the value of anti-β-actin as an internal control (fold change relative to control).

### 4.10. Statistical Analysis

Statistical analysis was performed using GraphPad Prism7. Data were presented as mean ± SEM. The difference between groups was determined by one-way ANOVA and Dunnett’s multiple comparisons test. *p* < 0.05 was considered to be statistically significant.

## 5. Conclusions

Overall, our findings demonstrated that Chr-A significantly suppressed the levels of inflammatory factors and inflammatory proteins on LPS-induced neuroinflammatory injury in vivo and in vitro. Furthermore, the mechanism of anti-neuroinflammation may be related to regulate the NLRP3/cleaved caspase-1 signaling pathway. However, whether Chr-A inhibited NLRP3/cleaved caspase-1 signaling pathway by directly interacting with NLRP3 was not discussed in this study. Thus, further experiments are still needed to reveal the in-depth mechanism. In summary, the results of the study showed that Chr-A might be a promising therapeutic candidate for neuroinflammation and might provide new inspiration in the development of therapeutic approaches for the treatment of CNS diseases, including stroke, traumatic brain injury and neurodegenerative disease.

## Data Availability

Not applicable.
